# Assessment of urinary pharmacokinetic and pharmacodynamic profiles of faropenem against extended-spectrum *β*-lactamase-producing *Escherichia coli* with canine *ex vivo* modelling: a pilot study

**DOI:** 10.1099/acmi.0.000004

**Published:** 2019-03-20

**Authors:** Kazuki Harada, Takae Shimizu, Naoki Miyashita, Yoshiaki Hikasa

**Affiliations:** 1 Department of Veterinary Internal Medicine, Tottori University, Minami 4-101, Koyama-Cho, Tottori 680-8553, Japan

**Keywords:** dogs, urinary tract infection, faropenem, extended-spectrum *β*-lactamase-producing bacteria, *ex vivo* model

## Abstract

This study was carried out to investigate the urinary pharmacokinetics and pharmacodynamics of faropenem administered orally at 5 mg kg^−1^ in six healthy dogs to assess the efficacy of the drug for canine urinary tract infections (UTIs) with extended-spectrum *β*-lactamase (ESBL)-producing bacteria. Six strains of ESBL-producing *Escherichia coli* (ESBL-EC) with the following faropenem minimum inhibitory concentrations (MICs) were used: 1 µg ml^−1^ (*n*=2), 2 µg ml^−1^ (*n*=2), 4 µg ml^−1^ (*n*=1) and 16 µg ml^−1^ (*n*=1). Urine samples were obtained every 4 h for the first 12 h after administration to measure urinary drug concentration and urinary bactericidal titres (UBTs). Both the urine concentration of faropenem and the UBTs for all tested strains peaked at 0–4 h after administration, and decreased markedly at 8–12 h. The mean urinary concentration of faropenem at 8–12 h (23±5.2 µg ml^−1^) exceeded the MIC of 1 µg ml^−1^ by fourfold, which is required to inhibit the growth of 90  % of ESBL-EC. These findings indicate that faropenem administered twice daily at a dose of 5 mg kg^−1^ is acceptable for the treatment of most dogs with ESBL-EC-related UTIs.

## Introduction

Bacterial urinary tract infections (UTIs) are common infectious diseases in dogs. Most UTIs can be managed successfully with appropriate antibiotic treatment; however, bacterial resistance as well as compromised host defence mechanisms can result in persistent/recurrent infections [1].

The emergence of extended-spectrum *β*-lactamase (ESBL)-producing bacteria in companion animals with UTIs is of great concern worldwide [2, 3]. Although ESBLs are usually involved in resistance to oxyimino-cephalosporins, penicillins and narrow-spectrum cephalosporins, ESBL-producing bacteria are often resistant to other classes of antimicrobials [4]. These multidrug-resistant phenotypes of ESBL-producing bacteria have major implications for the selection of adequate empirical therapy regimens [4].

*Escherichia coli* represents the most common bacterium causing canine UTIs, although a wide range of Gram-negative and Gram-positive bacteria can be detected as the causative organisms [5, 6]. We previously reported the high *in vitro* efficacy of several antimicrobials, including piperacillin/tazobactam, cefmetazole, amikacin, fosfomycin and faropenem, against ESBL-producing *E. coli* (ESBL-EC) isolates from companion animals [7]. Of these antimicrobials, faropenem is representative of the penem class and exhibits high bactericidal activity [8] and stability to hydrolysis by ESBLs [9, 10]; additionally, it is very safe to use in dogs [11, 12]. These findings indicate that faropenem may be a promising candidate antimicrobial for canine UTIs with ESBL-producing bacteria. However, the urinary pharmacokinetic/pharmacodynamic profile, which is essential for assessment of the treatment efficacy of antimicrobials for UTIs [13–15], remains to be investigated.

In the present study, we used liquid chromatography/mass spectrometry (LC-MS) to investigate the urinary pharmacokinetics of faropenem in dogs. We also measured the urinary bactericidal titres (UBTs) against ESBL-EC strains from canine UTIs to assess the urinary pharmacodynamics of this drug.

## Methods

### Sampling of urine from dogs treated with faropenem

Six beagle dogs (four male, two female; mean age and weight, 6.3±3.7 years and 12.4±1.18 kg, respectively) were purchased from Kitayama Labes Co. Ltd (Nagano, Japan). Prior to this study, all dogs were confirmed to be clinically healthy based on a physical examination, complete blood count, biochemical blood test and urinalysis. A balloon catheter was placed in the urinary bladder of each dog to allow urine collection. The dogs were administered faropenem (Farom Dry Syrup for Pediatric; Maruho Co. Ltd, Osaka, Japan) orally at a dose of 5 mg kg^−1^ body weight. Whole urine was obtained via a catheter at 4, 8 and 12 h after administration. The samples were sterilized by using 0.22 µm pore size filters (Starlab Scientific Co. Ltd, Shaanxi, People’s Republic of China) and stored at −80 °C until analysis.

### Measurement of urine faropenem concentration with LC-MS

Reference standard faropenem and cephalexin as the internal standard were separately dissolved in acetonitrile and then diluted with ultrapure water. LC-MS was carried out with a high-performance liquid chromatograph (LC-10AT; Shimadzu Co. Ltd, Kyoto, Japan). The mass spectra of faropenem and cephalexin were represented by peaks at *m/z* 308.05 and 348.10, respectively. The compounds were separated on a 2.1 mm internal diameter ×100 mm length 3 µm analytical column operated at 40 °C (Mastro C18; Shimadzu GLC Ltd, Tokyo, Japan). The mobile phase comprised 0.1 % formic acid aqueous solution and acetonitrile, and the flow rate was 0.2 ml min^−1^. The injection volume was 0.1 µl. Standard samples for the creation of a calibration curve were prepared with a blank urine matrix spiked with six concentrations of faropenem (1, 5, 10, 50, 100 and 500 µg ml^−1^). Standard and dog urine samples (50 µl) were mixed with 100 µg ml^−1^ of cephalexin (50 µl) as the internal standard and methanol (400 µl). After centrifugation at 12 000 r.p.m. for 5 min, the supernatants were harvested and then diluted 10-fold with ultrapure water for analysis. The validity of the LS-MS assay was verified according to the guidelines provided by the US Food and Drug Administration [16].

The urinary excretion (%) was calculated by dividing the excretion amount (mg) [i.e. a multiplication of the urine concentration (µg ml^−1^) by the urine volume (ml) in each time period] by the total dosage amount of faropenem (mg).

### Test organisms

The six ESBL-EC strains (strains ES-EC1–ES-EC6) from dogs with UTIs were selected from our collection [7] and used as representative faropenem-susceptible or -resistant ES-EC in this study. The faropenem minimum inhibitory concentrations (MICs) of these strains were determined according to the Clinical and Laboratory Standards Institute guidelines and categorized as in our previous study [7]: strains ES-EC1 and ES-EC2 (MIC: 1 µg ml^−1^, susceptible); strains ES-EC3 and ES-EC4 (MIC: 2 µg ml^−1^, susceptible); strain ES-EC5 (MIC: 4 µg ml^−1^, resistant); and strain ES-EC6 (MIC: 16 µg ml^−1^, resistant).

### Determination of urinary bactericidal titre

The UBTs corresponding to the maximal dilution titre of urine allowing bactericidal activity for each strain were determined by using each dog’s urine as described previously [15, 17]. Each well of the microplates contained 100 µl of the logarithmic serial twofold dilution ranging from 1 : 2 to 1 : 1024 by mixing an equal volume of the urine sample obtained every 4 h after administration and the individual dog’s antimicrobial-free urine obtained prior to drug administration. Subsequently, the tested organisms were inoculated with a final concentration of approximately 5×10^5^ c.f.u. ml^−1^. The bacterial number of inoculation was estimated based on the turbidity of the inoculation, and then quantified by the standard plate count method. Inoculated plates containing antibiotic-free urine samples (control) and 10 serially diluted urine samples obtained at 4, 8 and 12 h after administration were prepared for each dog and then incubated at 35 °C for 18 h. The subcultured urine was transferred to antimicrobial-free agar and then incubated at 35 °C overnight. The number of colonies grown was used to determine the bactericidal endpoint. The UBT was defined as a ≥99.9 % reduction of the initially inoculated colony counts. A UBT of 0 was defined as no bactericidal activity and a UBT of 1 was assigned when only undiluted urine displayed bactericidal activity. UBTs were transformed into ordinal data and described with reciprocal numbers [14, 15]. Simultaneously with UBT determination, it was confirmed that there was no bacterial growth in any of the urine samples without an inoculation of the tested organisms.

In addition, we calculated the geometric mean values of UBTs determined by using the urine of the six dog. For example, the UBTs of the six dogs’ urine for one strain were determined to be: 1 (2^0^), 16 (2^4^), 8 (2^3^), 16 (2^4^), 32 (2^5^) and 32 (2^5^). In this case, the geometric mean value of the UBT is 11.3, which is derived from 2^[(0+4+3+4+5+5)/6]^.

### Statistical analysis

Repeated analysis of variance with Bonferroni correction was used to compare urine concentrations between the collection time periods after the values below the minimum quantification level were replaced with zero. The geometric mean values of the UBT for each strain were compared between the collection time periods by standard one-way analysis of variance with Bonferroni’s post hoc comparison test. A *P*-value of <0.05 was considered significant for all analyses.

## Results

### Safety and laboratory test results

No adverse effects were observed in any dogs during the test period. The results of the physical examination, complete blood count and biochemical blood test displayed no clinically relevant changes.

### Urine concentration and urinary excretion of faropenem

**Fig. 1.**
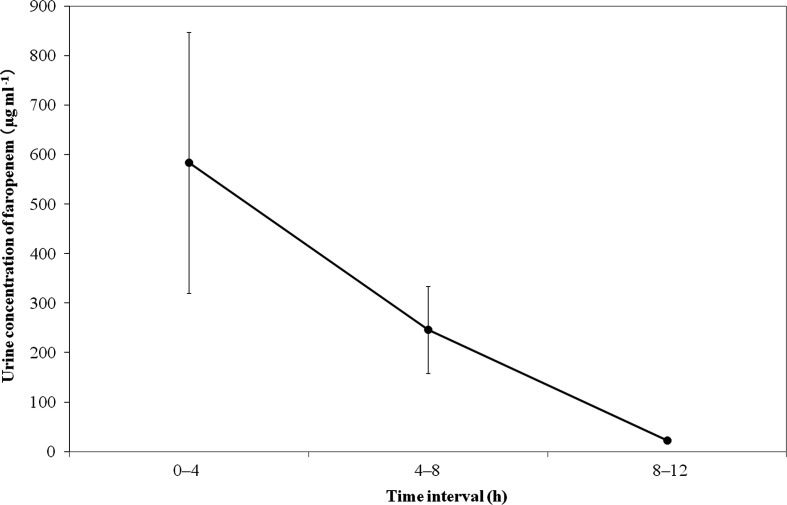
Concentration of faropenem in dogs’ urine after administration at a dose of 5 mg kg^−1^ body weight (mean±sem, *n*=6).

The LC-MS assay showed a minimum quantification level at 10 ng ml^−1^ for faropenem in dog urine. The urinary concentration (mean±se) peaked 0 to 4 h after administration (584±263 µg ml^−1^) and then decreased to 246±88 µg ml^−1^ at 4 to 8 h and 23±5.2 µg ml^−1^ at 8 to 12 h (Fig. 1). A significant difference in the urine concentration was observed between 0 to 4 h and 8 to 12 h (*P*<0.05). All urine samples collected prior to drug administration had no detectable drug. The urinary excretion (mean±se) was 25.3±5.9 % at 0 to 4 h and then remained almost unchanged from 4 to 8 h (34.3±7.4 %) to 8 to 12 h (35.5±7.2 %).

### UBTs

The temporal changes in the median UBTs for each strain are shown in Table 1. In all tested ESBL-EC strains, the geometric means of the faropenem UBTs peaked at 0 to 4 h and then decreased at 4 to 8 h and 8 to 12 h. The UBTs at 8–12 h were significantly lower than those at 0–4 h and 4–8 h (*P*<0.05).

**Table 1.** Geometric means (−GSD, +GSD) of the urinary bactericidal titres (UBTs) of faropenem (5 mg kg^−1^ body weight) for the six extended-spectrum *β*-lactamase-producing *Escherichia coli* strains tested in this study

Strains	MIC	UBTs at each time interval (h)*		
	(µg ml^−1^)	0–4	4–8	8–12
ES-EC1	1	287.4 (127.8, 646.1)^a^	128.0 (53.3, 307.6)^b^	11.3 (3.1, 41.4)^a,b^
ES-EC2	1	228.1 (101.4, 512.8)^a^	181.0 (77.5, 423.1)^b^	14.3 (3.7, 54.7)^a,b^
ES-EC3	2	128.0 (48.0, 341.1)^a^	50.8 (21.9, 117.6)^b^	3.6 (1.0, 12.7)^a,b^
ES-EC4	2	256.0 (96.1, 682.3)^a^	90.5 (61.9, 132.3)^b^	7.13 (2.0, 25.4)^a,b^
ES-EC5	4	40.3 (28.2, 57.7)^a^	20.2 (8.7, 46.7)^b^	2.8 (1.2, 6.6)^a,b^
ES-EC6	16	32.0 (17.2, 59.5)^a^	16.0 (8.6, 29.7)^b^	1.6 (0.9, 2.8)^a,b^

*There were significant differences between values with the same letters within a row (*P*<0.05).

GSD, geometric standard deviation.

## Discussion

Although faropenem shows excellent *in vitro* antimicrobial activity against ESBL-producing bacteria [7], its efficacy for canine UTIs with these bacteria has not been previously assessed. The urinary drug concentration is correlated with the antibacterial activity in UTIs; however, the activity of an antimicrobial drug can be affected by the biological urine matrix [18]. In this study, we investigated both the concentration of faropenem in urine and the UBTs in dogs after the administration of faropenem to assess the efficacy of the drug. To the best of our knowledge, this is the first report to investigate the urinary pharmacokinetics and pharmacodynamics of faropenem in dogs.

The present study demonstrated that in dogs approximately one-third of the dose of faropenem is excreted in urine during the 12 h after oral administration. This urinary excretion of faropenem in dogs is higher than that in humans: the urinary excretion of the drug after oral administration at a 300 mg dose in human patients (approximately 5 mg kg^−1^ body weight) was merely 14 to 20 % [10]. These findings imply that this drug is more suitable for the treatment of UTIs in dogs than in humans, possibly because of the differences in absorption, distribution, metabolism and elimination between dogs and humans.

In this study, we found a high concentration of faropenem at an early stage after administration and an extremely low concentration of faropenem in dog urine at 8–12 h after administration. The UBTs (which can serve as a pharmacokinetic/pharmacodynamic assessment parameter for antimicrobial agents in the urine [14, 15]) of faropenem in all tested strains fluctuated in close alignment with the concentration of the drug in the urine during the test period. These findings indicate that the urinary excretion and bactericidal activity of faropenem practically expire at 12 h after oral administration in dogs. This urinary pharmacokinetic and pharmacodynamic properties of faropenem differ greatly from those of once-a-day fluoroquinolones, which can maintain high urinary concentrations until 24 h after oral administration [15, 19, 20].

The efficacy of time-dependent antibiotics, including faropenem, is predicted by the time that the drug concentration exceeds the two- to fourfold MIC of pathogens [21]. Thus, time-dependent antibiotics can be optimized by using dosing strategies that maximize the duration of drug exposure [22]. In this study, we adopted 5 mg kg^−1^ body weight for the administration of faropenem per dose, according to the dosage established for humans [10]. It was found that the mean urinary concentration of faropenem at 8–12 h was 23 µg ml^−1^, indicating that twice-daily faropenem at a dose of 5 mg kg^−1^ has therapeutic efficacy for UTIs by ES-EC strains with MICs of ≤4 µg ml^−1^. This result implies that most ESBL-EC-related UTIs in dogs can be theoretically treated with faropenem at an oral dose of 5 mg kg^−1^, because 90 % of ES-EC strains from dogs and cats have an MIC of ≤1 µg ml^−1^ [7]. On the other hand, the same dosing strategy might fail to treat UTIs by strains with MICs of ≥8 µg ml^−1^. In fact, the UBTs of strain ES-EC6 (faropenem MIC of 16 µg ml^−1^) were lower than those of the remaining tested strains (MICs of ≤4 µg ml^−1^) during the test periods. These findings suggest that thrice-daily rather than twice-daily administration of faropenem is preferable for the treatment of UTIs by strains with higher MICs.

There were several limitations in this study. Firstly, it was carried out as a pilot study by using a small number of dogs, and thus the present results might be somewhat biased. Secondly, only healthy experimental dogs were used in this study, although the pharmacokinetics of faropenem may possibly differ in dogs with UTIs or household dogs.

Nevertheless, we determined the UBTs and related parameters of faropenem in dogs to assess the efficacy of this drug against canine UTIs with ESBL-producing bacteria. Based on the urinary pharmacokinetics and UBTs, faropenem administered twice daily at a dose of 5 mg kg^−1^ is acceptable for the treatment of most dogs with ES-EC-related UTIs. We strongly believe that the present study serves as a basis for the clinical application of faropenem for ESBL-producing bacteria-related UTIs in dogs.

## References

[1] Smee N, Loyd K, Grauer GF (2013). UTIs in small animal patients: part 2: diagnosis, treatment, and complications. J Am Anim Hosp Assoc.

[2] Ewers C, Grobbel M, Stamm I, Kopp PA, Diehl I (2010). Emergence of human pandemic O25:H4-ST131 CTX-M-15 extended-spectrum-beta-lactamase-producing *Escherichia coli* among companion animals. J Antimicrob Chemother.

[3] Marques C, Belas A, Franco A, Aboim C, Gama LT (2018). Increase in antimicrobial resistance and emergence of major international high-risk clonal lineages in dogs and cats with urinary tract infection: 16 year retrospective study. J Antimicrob Chemother.

[4] Pitout JD, Laupland KB (2008). Extended-spectrum *β*-lactamase-producing *Enterobacteriaceae*: an emerging public-health concern. Lancet Infect Dis.

[5] Ling GV, Norris CR, Franti CE, Eisele PH, Johnson DL (2001). Interrelations of organism prevalence, specimen collection method, and host age, sex, and breed among 8,354 canine urinary tract infections (1969–1995). J Vet Intern Med.

[6] Smee N, Loyd K, Grauer G (2013). UTIs in small animal patients: part 1: etiology and pathogenesis. J Am Anim Hosp Assoc.

[7] Shimizu T, Harada K, Tsuyuki Y, Kimura Y, Miyamoto T (2017). *In vitro* efficacy of 16 antimicrobial drugs against a large collection of *β*-lactamase-producing isolates of extraintestinal pathogenic *Escherichia coli* from dogs and cats. J Med Microbiol.

[8] Matsuzaki K, Nishiyama T, Hasegawa M, Kobayashi I, Kaneko A (1999). *In vitro* bactericidal activities of new oral penem, faropenem against the various clinical isolates. Jpn J Antibiot.

[9] Dalhoff A, Nasu T, Okamoto K (2003). Beta-lactamase stability of faropenem. Chemotherapy.

[10] Hamilton-Miller JM (2003). Chemical and microbiologic aspects of penems, a distinct class of beta-lactams: focus on faropenem. Pharmacotherapy.

[11] Okamoto M, Ochiai T, Ichiki T (1998). The effects on kidneys of dogs after a single or repeated dosing of faropenem sodium. Jpn Pharmacol Ther.

[12] Faqi AS, Lanphear C, Gill S, Colagiovanni DB (2010). Juvenile toxicity study of faropenem medoxomil in beagle puppies. Reprod Toxicol.

[13] Wagenlehner FM, Naber KG (2004). Antibiotic treatment for urinary tract infections: pharmacokinetic/pharmacodynamic principles. Expert Rev Anti Infect Ther.

[14] Wagenlehner FM, Wagenlehner C, Redman R, Weidner W, Naber KG (2009). Urinary bactericidal activity of doripenem versus that of levofloxacin in patients with complicated urinary tract infections or pyelonephritis. Antimicrob Agents Chemother.

[15] Shimizu T, Harada K, Manabe S, Tsukamoto T, Ito N (2017). Assessment of urinary pharmacokinetics and pharmacodynamics of orbifloxacin in healthy dogs with *ex vivo* modelling. J Med Microbiol.

[16] Food and Drug Administration (2001). Guidance for industry, Bioanalytical method validation. http://www.fda.gov/downloads/Drugs/Guidance/ucm070107.pdf#search=%27Bioanalytical+Method+Validation%2C+Center+for+Drug+Evaluation+and+Research.%27.

[17] Well M, Naber KG, Kinzig-Schippers M, Sörgel F (1998). Urinary bactericidal activity and pharmacokinetics of enoxacin versus norfloxacin and ciprofloxacin in healthy volunteers after a single oral dose. Int J Antimicrob Agents.

[18] So W, Crandon JL, Nicolau DP (2015). Effects of urine matrix and pH on the potency of delafloxacin and ciprofloxacin against urogenic *Escherichia coli* and *Klebsiella pneumoniae*. J Urol.

[19] Monlouis JD, De Jong A, Limet A, Richez P (1997). Plasma pharmacokinetics and urine concentrations of enrofloxacin after oral administration of enrofloxacin in dogs. J Vet Pharmacol Ther.

[20] Daniels JB, Tracy G, Irom SJ, Lakritz J (2014). Fluoroquinolone levels in healthy dog urine following a 20-mg/kg oral dose of enrofloxacin exceed mutant prevention concentration targets against *Escherichia coli* isolated from canine urinary tract infections. J Vet Pharmacol Ther.

[21] Barger A, Fuhst C, Wiedemann B (2003). Pharmacological indices in antibiotic therapy. J Antimicrob Chemother.

[22] Levison ME, Levison JH (2009). Pharmacokinetics and pharmacodynamics of antibacterial agents. Infect Dis Clin North Am.

